# 

**Published:** 1896-02-08

**Authors:** 


**Notices of Births, Deaths, and Marriages** are inserted in this column at a charge of 2s. 6d. for an announcement not exceeding four lines.

**Notice to Advertisers and Subscribers.**

All Advertisements, Orders for Copies of The Hospital and Business Communications should be addressed to **THE MANAGER, (Not to The Editor)**, The Hospital, 428, Strand, London, W.C.

The Cost of Subscription, post free, is as follows:—Per week, 2½d.; per quarter, 2s. 8d.; per half-year, 5s. 3d.; per annum, 10s. 6d. Foreign:—Per Annum, 15s. 2d.; payable, in all cases, in advance.

### NOTICE TO CORRESPONDENTS.

All MS., Letters, Books for Review, and other matters intended for the Editor should be addressed The Editor, The Lodge, Porchester Square, London, W.

The Editor cannot undertake to return rejected MS., even when accompanied by stamped directed envelope.

**"Burdett's Hospitals and Charities."**

Sixth year of publication. 950 pp. Scarlet cloth, gilt lettered. Price 5s. A most complete guide to British, Colonial, Indian, and American Hospitals, Dispensaries, Nursing and Convalescent Institutions, and Asylums. A few complete sets of the preceding five issues of the "Annual" may still be obtained on application to the Publishers, The Scientific Press (Limited), 428, Strand, London, W.C.

**NOTICE.—Vol. XVIII. of "The Hospital" (April to September, 1895), in handsome cloth binding, is on sale at the Office of this Journal. Price 7s. 6d. Cases for binding the Numbers contained in this Volume, 1s. 6d. Index to Vol. XVIII., 2½d., post free. The Monthly Part for February is now ready, price 6d., by post, 8d.**

### THE STUDENT OF MEDICINE.

### APPOINTMENTS.

J. Atkinson, M.B., C.M.Edin., appointed Medical Officer to the Felsted School, Essex. A. G. R. Cameron, M.B., B.S., M.R.C.P., L.R.C.P., D.P.H.Camb., appointed Assistant Medical Officer to the Small-pox Hospital Ships of the Metropolitan Asylums Board. R. W. Dodgson, M.R.C.S., L.R.C.P., appointed House Physician to St. Mary's Hospital, Paddington, W. M. G. Dundas, M.R.C.S.Eng., L.S.A., appointed Medical Officer of Health to the Mitford and Launditch Rural District Council. P. Fraser, M.D.Edin., B.Sc., appointed Medical Officer of Health for the North Wales United District. A. C. Key, M.D.St.And., M.R.C.P.Edin., L.S.A.Lond., appointed Honorary Physician to the Royal Society of Musicians of Great Britain. E. T. Larkham, M.R.C.S., L.R.C.P.Lond., D.P.H., appointed Medical Officer for the Munslow District of the Ludlow Union. J. H. McAuley, L.R.C.P., L.R.C.S.Edin., appointed Medical Officer for the Summerhill Dispensary District of the North Dublin Union. Dr. A. T. Messiter, appointed Medical Officer of Health to the Isle of Axholm Rural District Council. P. W. G. Nunn, L.R.C.P.Lond., M.R.C.S., appointed Medical Officer of Health to the Pokesdown Urban District Council. E. L. Pridmore, M.B.Lond., M.R.C.S., appointed Medical Officer for the Weymouth District of the Weymouth Union. T. D. Savill, M.D.Lond., M.R.C.P.Lond., D.P.H.Camb., appointed Physician to the Hospital for Diseases of the Nervous System, Welbeck Street, London. C. A. Wickham, L.R.C.P., L.R.C.S.Edin., appointed Medical Officer for the Fourth District of the East Ashford Union. Dr. J. Wilson, appointed Medical Officer to the Enniskillen Workhouse Hospital. J. F. Wright, L.R.C.P.Lond., M.R.C.S.Eng., appointed Medical Officer for the Western Bolton District of the Bolton Union. Mrs. Flemming, M.D.Lond., appointed Medical Registrar to the Royal Free Hospital. Miss L. B. Aldrich-Blake, M.D., M.S., appointed Surgical Registrar to the Royal Free Hospital. W. H. Evans, M.D.Lond., F.R.C.S.Eng., appointed Assistant Surgeon, with charge of special department for Skin Diseases, to the Royal Free Hospital. R. H. P. Crawfurd, M.A., M.D.Oxon, M.R.C.P.Lond., appointed Assistant Physician to the Royal Free Hospital.

### VACANCIES.

### Resident Medical Officers.

Birmingham and Midland Eye Hospital, Church Street, Birmingham.—Assistant House Surgeon. Salary £50 per annum, with apartments and board. Applications to the Chairman of the Medical Board by February 13th.

Cardiff Union.—Assistant Medical Officer for the Workhouse Appointment for one year. Salary £100 per annum, with rations, apartments, attendance, and washing. Applications to Arthur J. Harris, Clerk, Queen's Chambers, Cardiff, by February 18th.

Chichester Infirmary.—House Surgeon. Salary £80 per annum, with board, lodging, and washing. Applications to the Secretary by February 17th.

General Infirmary and Dispensary, Doncaster.—Indoor Dispenser and Assistant to House Surgeon. No salary, but board, lodging, and washing provided. Applications to Joseph Clark, Honorary Secretary, by February 10th.

Queen's Jubilee Hospital, Earl's Court, S.W.—House Surgeon appointment for six months, but eligible for re-election. Salary at the rate of £100 per annum, and rooms adjoining the hospital. Applications to the Secretary by February 8th.

### Non-Resident Medical Officers.

Dental Hospital of London, Leicester Square.—Assistant Dental Surgeon. Applications to J. Francis Pink, Secretary, by March 9th.

Liverpool Royal Infirmary.—Honorary Assistant Physician. Applications to the Chairman of the Committee of the Royal Infirmary, Liverpool, by February 20th.

London Hospital, Whitechapel, E.—Two Surgeon Dentists. Applications to the House Governor by February 28th.

Middlesex Hospital Medical School.—Lecturer on Anatomy and a Lecturer on Physiology. Applications to the Dean of the Medical School, Cleveland Street, W., by February 17th.

St. Mark's Hospital, City Road, E.C.—Assistant Surgeon; must be Fellow of the Royal College of Surgeons. Applications to the Secretary, Mr. Arthur Leard, by February 15th.

### Royal College of Surgeons of England.

The following gentleman, having previously passed the necessary examinations, and having now attained the legal age of 25 years, was at the quarterly meeting of the Council on January 9th admitted a member of the college: J. W. Haines, M.B.Lond., L.R.C.P.Lond., St. Bartholomew's Hospital; diploma of member, dated July 30th, 1894.

The following gentleman, having previously passed the necessary examinations, and having conformed to the by-laws and regulations, was admitted a member of the college: C. W. Moorshead, L.S.A.Lond., Guy's Hospital.

A FEW COPIES STILL LEFT.

Sixth year of publication. 950 pp. Crown 8vo., red cloth, gilt, 5s.

BURDETT'S HOSPITAL AND CHARITIES ANNUAL:
Being the Year-Book of Philanthropy, 1895.
Edited by HENRY C. BURDETT,
Author of "Hospitals and Asylums of the World," "Cottage Hospitals," "Helps in Sickness and to Health," &c., &c.

"Requires no more than the name of its editor to warrant that a subject of great and growing importance is dealt with in a thoroughly satisfactory manner."—*The Times.*

London: THE SCIENTIFIC PRESS (LIMITED), 428, Strand, W.C.

BIRMINGHAM AND MIDLAND EYE HOSPITAL.

### THE 2/6 SERIES OF NURSING MANUALS.

**HOW TO BECOME A NURSE: AND HOW TO SUCCEED.** A complete guide to the nursing profession for those who wish to become Nurses, and a useful book of Reference for Nurses who have completed their training and seek employment. Compiled by HONNOR MORTEN, Author of "The Nurses' Dictionary," &c. Third and Revised Edition. Demy 8vo, 209 pp., profusely Illustrated with Copyright Portraits and Drawings.

"To those who are frequently appealed to by girls, or young women, as to the steps they should take to become nurses, this book of Miss Morten's must prove a perfect godsend."—*British Medical Journal.*

**THE NURSES' DICTIONARY OF MEDICAL TERMS AND NURSING TREATMENT.** Compiled for the use of Nurses, by HONNOR MORTEN. Containing descriptions of the principal Medical and Nursing Terms and Abbreviations, Instruments, Drugs, Diseases, Accidents, Treatments, Physiological Names, Operations, Foods, Appliances, &c., &c., encountered in the Ward or Sick-room. Third and Revised Edition (25th thousand). Demy 16mo, suitable for the apron pocket, handsomely bound in handsome leather, gilt, price 2s. 6d. net, or in cloth, price 2s.

"Should be at the disposal of every nurse."—*Birmingham Medical Review.*

**HOSPITAL SISTERS AND THEIR DUTIES.** By EVA C. LUCKES, Matron to the London Hospital. The favourable reception accorded to the first two editions of this useful and interesting work, and the regular demand for copies of it, encourage the publishers to place this third edition, greatly improved and enlarged, before the Institutions. The experience and position of its author entitle it to authority, and every Matron, Sister, or Nurse who aspires to become a Sister, should read the advice and information contained in its pages. Third Edition. Enlarged and partly rewritten, crown 8vo, cloth gilt, about 200 pp.

**MENTAL NURSING.** A Text-book specially designed for the instruction of Attendants on the Insane. By WILLIAM HARDING, M.D., M.R.C.P. The want of a complete book for the instruction of Asylum Attendants has been long felt, and it is with confidence that the publishers recommend this work to the managers of all Institutions for the Insane. Second Edition. Enlarged, demy 8vo, profusely illustrated, nearly 200 pp., cloth.

**SPINAL CURVATURE AND AWKWARD DEPORTMENT: Their Causes and Prevention in Children.** By Dr. GEORGE MULLER, Professor of Medicine and Orthopædics, Berlin. English Edition, edited and adapted by RICHARD GREENE, F.R.C.P. Crown 8vo, cloth gilt, with many Plates and Illustrations.

"Dr. Müller's little book is an able essay upon the effect produced by exercise, attitude, and movement upon the growth and development of the body. He further gives directions which any intelligent parent or teacher could carry out with the help of very simple apparatus, showing how to avoid or, in early cases, to cure certain malformations which, in the majority of cases, arise either from the neglect of very simple hygienic rules or from carelessness."—*Daily Chronicle.*

**THE MOTHER'S HELP AND GUIDE TO THE DOMESTIC MANAGEMENT OF CHILDREN.** By P. MURRAY BRAIDWOOD, M.D., formerly Senior Medical Officer to the Wirral Hospital for Sick Children. Crown 8vo, cloth gilt.

"The book is altogether admirable."—*Manchester Courier.*

London: THE SCIENTIFIC PRESS (Limited), 428, STRAND, W.C.

### "THE HOSPITAL"

AN INDEPENDENT JOURNAL OF

**The Medical Sciences**
AND
**Hospital Administration,**

WITH WHICH IS ISSUED WEEKLY

A SPECIAL

### SUPPLEMENT FOR NURSES.

PUBLISHED EVERY SATURDAY.
PRICE TWOPENCE.

Matrons, Sisters, or Nurses will find in The Hospital a chronicle of the latest Nursing News, Practical Articles of the highest value to those who wish to keep themselves abreast with the times, and brightly written articles from Nurses all over the world.

**SUBSCRIPTIONS CAN COMMENCE AT ANY TIME.**

### TERMS OF SUBSCRIPTION.

### WEEKLY ISSUE.

| | Great Britain. | Foreign and Colonial. |
|---|---|---|
| For One Year ... | 10s. 6d. | 15s. 2d. |
| For Six Months ... | 5s. 3d. | 7s. 7d. |
| For Three Months... | 2s. 8d. | 3s. 10d. |

### MONTHLY PARTS.

| | Post Free to any Part of the World. |
|---|---|
| For One Year ... | 8s. 6d. |
| For Six Months ... | 4s. 3d. |
| For Three Months ... | 2s. 2d. |

Postal Orders and Cheques should be made payable to "The Scientific Press, Limited."

N.B.—In order to prevent delay, all business communications should be addressed to "The Manager" only, and not to any person individually.

### CHANGE OF ADDRESS.

In event of change of address, notice should be forwarded to the Publishers by the Saturday previous to date of publication, and the old address should also be given.

"The Hospital" can be obtained of all Booksellers and Newsagents in the United Kingdom, and at all the Railway Stalls, of Messrs. W. H. Smith and Son; and also from the following Agents in the Colonies and Abroad:—

Paris: Librairie Galignani, 224, Rue de Rivoli.
Berlin: Messrs. A. Asher and Co.
Leipsig: Mr. F. A. Brockhaus.
New York: The International News Co.
Montreal: The Montreal News Co.
Toronto: The Toronto News Co.
Melbourne, Sydney, Adelaide, and New Zealand: Messrs. Gordon and Gotch.
India: At all the Railway Bookstalls and Agencies of Messrs. A. H. Wheeler and Co.
South Africa: Messrs. Juta, Cape Town.

Publishing Offices: 428, Strand, London, W.C.

*In the Press.* *Price 5s.*

### BURDETT'S OFFICIAL NURSING DIRECTORY

**A DIRECTORY, NOT A REGISTER.**

**THE HOSPITAL states: "It is well to bear in mind the difference and distinction which exists between a register and a directory. A register implies rights, and suggests that those who are not upon it are in some way or another inferior beings. But a directory is another matter. A directory gives as rights; insertion in it implies no adherence to any particular party or any particular opinion, and in no way interferes with entry on the register of any association, or the list of any institute, to which the individual may belong. All that a directory professes to do is to give information. The 'Medical Register' is not a mere list of qualified medical men; it is admission to the Register, which itself makes him qualified. For years, perhaps even now, 'Churchill's Medical Directory' contained the names of medical men who, so far as public appointments were concerned, were unqualified; it also contains the names of homœopathists, whom four out of every five medical men will not meet; and it certainly contains the names of many for whose moral character we should not like to vouch. But it gives full and accurate information about them all, and for the sake of that information it is constantly consulted both by the public and the doctors."**

THE SCIENTIFIC PRESS (Lim.), 428, Strand, London, W.C.

